# Yoga for Cancer Survivors (YOCAS): A Systematic Review of the YOCAS Program’s Impact on Physical and Psychological Well-Being

**DOI:** 10.7759/cureus.71857

**Published:** 2024-10-19

**Authors:** Selvaraj Giridharan, Soni Soumian, Jawaher Ansari

**Affiliations:** 1 Oncology, Tawam Hospital, Al Ain, ARE; 2 General Surgery, Tawam Hospital, Al Ain, ARE

**Keywords:** cancer, cancer-related fatigue, cancer survivorship, cognitive function, complementary therapies, musculoskeletal symptoms, sleep quality, yoga

## Abstract

Cancer survivors frequently experience prolonged physical and psychological symptoms including cancer-related fatigue (CRF), sleep disturbances, cognitive impairment, and musculoskeletal pain. Conventional treatments for these symptoms have demonstrated limited efficacy, emphasising the need for complementary therapies. The Yoga for Cancer Survivors (YOCAS) program is a structured mind-body intervention designed to address these challenges. This systematic review evaluated the efficacy of YOCAS in managing CRF, sleep quality, cognitive function, and musculoskeletal symptoms in cancer survivors using randomised controlled trials (RCTs). A comprehensive search was conducted across the Google Scholar, Scopus, Cochrane Library, and PubMed databases for RCTs published between January 2000 and September 2024. Eligible studies included adult cancer survivors who had completed primary treatment and compared YOCAS interventions to control groups. The primary outcomes were cancer-related fatigue, sleep quality, cognitive function, and musculoskeletal symptoms. The risk of bias was evaluated using the Cochrane Risk of Bias Tool, and the findings were synthesised. Six RCTs, involving 1,717 participants, met the inclusion criteria. The YOCAS program demonstrated significant improvements in the reduction of cancer-related fatigue and sleep quality. Cognitive function and memory were improved, particularly among breast cancer survivors, with reduced musculoskeletal pain reported in participants undergoing hormonal therapy. Despite variations in study design, the risk of bias was generally low. ​​​​​​​The YOCAS program effectively reduced cancer-related fatigue, improved sleep quality, and addressed the cognitive and musculoskeletal symptoms in cancer survivors. Given its low risk and broad applicability, YOCAS shows promise as a complementary therapy for cancer survivorship care. Future research should focus on the long-term sustainability of these benefits and explore the impact of the program across diverse cancer populations.

## Introduction and background

Cancer remains one of the leading causes of mortality globally, with the incidence of cancer and cancer-related deaths increasing significantly in recent years. In 2019, there were 23.6 million new cancer cases and 10 million cancer-related deaths worldwide, reflecting a 26.3% increase in the number of new cases since 2010 [1,2]. Advancements in medical treatment, early detection, and precision cancer medicine have improved the survival rate and quality of life of numerous patients [3,4]. However, the growing population of survivors faces long-term physical and psychological challenges that significantly impact their wellbeing [5]. According to the US National Coalition for Cancer Survivorship, a cancer survivor is defined as an individual diagnosed with cancer, whether still in treatment or cancer-free, and their care needs extend well beyond the disease itself [6,7].

Cancer survivorship is frequently accompanied by a range of persistent symptoms including cancer-related fatigue (CRF), sleep disturbances, cognitive impairments, and musculoskeletal pain [8-10]. These symptoms can persist for months or even years following treatment, significantly affecting daily functioning and quality of life (QoL). Despite the progress in cancer treatment, managing these long-term effects remains a challenge. Standard interventions such as pharmacological treatments often offer limited relief and are associated with undesirable side effects, highlighting the necessity for alternative approaches [11].

Traditional medical treatments for symptoms such as pain, fatigue, and sleep disorders can be efficacious, but are often insufficient in isolation, particularly when considering their limitations in efficacy and potential side effects. Consequently, complementary therapies have garnered increasing attention in addressing the holistic needs of cancer survivors [12-16]. Yoga has emerged as one of the most promising complementary therapies because of its dual focus on physical and mental well-being. Research has demonstrated that yoga can reduce stress, improve mood, and enhance physical functioning, rendering it a valuable intervention for survivors of cancer [17-20]. 

Yoga, an ancient mind-body practice, incorporates physical posture, breathing exercises, and meditation. It is widely recognised for its ability to improve both physical and psychological health [21]. Among the various yoga interventions, the Yoga for Cancer Survivors (YOCAS) program has gained prominence as a structured and standardised form of yoga designed specifically for cancer survivors. The program, developed by the University of Rochester Medical Center, is a standardised intervention aimed at improving the physical and psychological well-being of cancer survivors. It incorporates Gentle Hatha and Restorative Yoga to address common survivorship issues, such as CRF, sleep disturbances, stress, and pain. Sessions were conducted twice weekly over four weeks, each lasting 75 minutes, in community-based settings, such as yoga studios or oncology centres, with groups of 10 to 15 participants.

The program’s three core components - movement (asana), breathing (pranayama), and awareness (mindfulness meditation) - are delivered by certified instructors who undergo standardised training to ensure consistency and accessibility for all participants. The focus on restorative poses aims to improve sleep and promote relaxation, whereas mindfulness and breathing exercises enhance overall well-being. Preliminary studies suggest that yoga interventions, such as YOCAS, can improve cancer-related fatigue, sleep quality, cognitive function, and physical symptoms, such as pain and stiffness. However, evidence supporting these benefits is still evolving, and a comprehensive review of the research is necessary to better understand the program’s effectiveness.

Several clinical trials have investigated the impact of YOCAS on cancer-related symptoms; however, the results have yet to be systematically reviewed. This systematic review aimed to synthesise findings from clinical trials assessing the effects of the YOCAS program on key cancer-related outcomes. Specifically, this review will evaluate the program’s impact on CRF, sleep quality, cognitive function, and musculoskeletal symptoms. By consolidating the available evidence, this review provides clearer insights into the role of YOCAS in improving the quality of life of cancer survivors and informs future research and clinical practice.

## Review

Methods

This systematic review was conducted to evaluate the effectiveness of a Yoga for Cancer Survivors (YOCAS) program on cancer-related fatigue (CRF), sleep quality, cognitive function, and musculoskeletal symptoms in cancer survivors. The Preferred Reporting Items for Systematic Reviews and Meta-Analyses (PRISMA) guidelines were adhered to throughout the review process to ensure transparency and completeness [22]

Eligibility Criteria

This review included only randomised controlled trials (RCTs) to ensure high-quality evidence. Eligible studies focused on adult cancer survivors (aged ≥ 18 years) who had completed primary cancer treatment and evaluated the effects of the YOCAS program on fatigue, sleep, cognitive function, or musculoskeletal symptoms. Studies were required to compare YOCAS to a control group (e.g. usual care or waitlist control) and to report at least one relevant outcome. Only studies published in English were included.

Search Strategy

A comprehensive literature search was conducted using four major databases: Google Scholar, Scopus, the Cochrane Library, and PubMed. The search included studies published between January 2000 and September 2024. The following search terms were employed individually and in combination: "Yoga for Cancer Survivors," "YOCAS," "cancer-related fatigue," "sleep quality," "cognitive function," "musculoskeletal symptoms," and "randomized controlled trials." The search strategy was tailored to each database, utilising MeSH terms in PubMed and equivalent subject headings or keywords in the other databases. Boolean operators (AND, OR) were used to refine the search and ensure that relevant studies were identified.

Study Selection

All retrieved articles were initially screened based on their titles and abstracts. Full-text articles were obtained for studies that met the eligibility criteria or that appeared to meet the criteria based on the abstract. Two independent reviewers conducted the selection process, and disagreements were resolved through discussion or consultation with a third reviewer.

Data Extraction

Two reviewers independently extracted the data using a standardised form. The information collected included study design, participant characteristics (e.g. age, cancer type), details of the YOCAS intervention (e.g. duration and frequency), control group interventions, primary and secondary outcomes (e.g. fatigue, sleep quality, cognitive function), sample sizes, and statistical data such as effect sizes, confidence intervals, and significance of outcomes.

Risk of Bias Assessment

Two reviewers independently assessed the risk of bias for each study using the Cochrane Risk of Bias Tool [23]. The following domains were evaluated: random sequence generation, allocation concealment, blinding of participants and personnel, blinding of outcome assessment, incomplete outcome data, selective reporting, and other potential biases. Discrepancies in assessments were resolved by discussion or consultation with a third reviewer. 

Data Synthesis

Due to the variability in the reporting of outcomes and the interventions, a narrative synthesis was performed to summarise the findings from the included studies. Primary outcomes (CRF, sleep quality, cognitive function, and musculoskeletal symptoms) were reported. Quantitative synthesis (meta-analysis) was considered, but not performed.

Results

**Figure 1:**
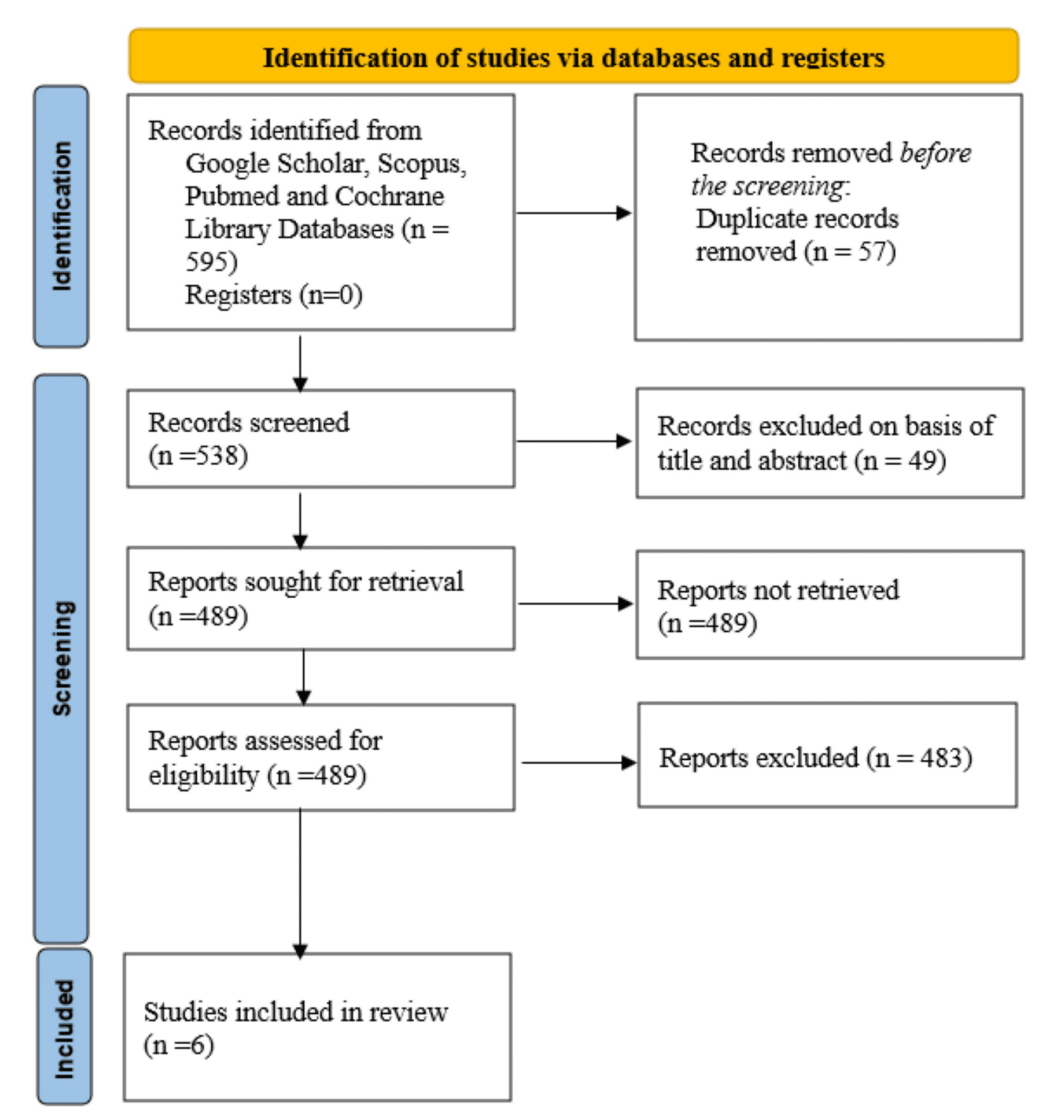
Summarized search strategy (Preferred Reporting Items for Systematic Reviews and Meta-Analyses flow diagram)

The search identified 587 records across all databases, and 57 duplicates were removed, leaving 530 articles for screening. After excluding 505 records that did not meet the inclusion criteria, 25 reports were reviewed in full, resulting in the inclusion of six studies in the final analysis (Figure 1). 

The following sections summarise the key findings across these trials, organised by the primary outcomes of cancer-related fatigue (CRF), sleep quality, cognitive function and memory, and musculoskeletal symptoms [24-29]. The study characteristics are summarised in Table 1.

**Table 1:** Characteristics of Included Studies Evaluating the YOCAS Program in Cancer Survivors PSQI: Pittsburgh Sleep Quality Index, MDASI: MD Anderson Symptom Inventory, MFSI-SF: Multidimensional fatigue symptom inventory – Short Form, FACIT-F: Functional Assessment of Chronic Illness Therapy with Fatigue Subscale, CRF: Cancer-related fatigue, QoL: Quality of Life, YC: YOCAS group, SC: Standard Care Group, YOCAS: Yoga for Cancer Survivors.

Author(s)	Cancer Type	Sample Size	Measurements	Tools used	Outcomes	
					Primary	Secondary
Mustian et al. [29]	Various (75% Breast)	YC: 206 SC:204	Sleep quality	PSQI, Actigraphy	Significant improvement in global sleep quality (P<0.01)	Daytime dysfunction (P<0.01) Subjective sleep quality (P<0.05) Sleep medication use (P<0.05) Sleep latency (P<0.01), Sleep duration (P<0 .05) Sleep efficiency (P<0.01) Sleep disturbances (P<0.05)
Sprod et al. [28]	Various (65% Breast)	YC: 53 SC:44 >60 yr olds	Cancer-related fatigue (CRF)	MFSI-SF, Clinical symptom inventory	Lower level of CRF (P=0 .03), Less physical fatigue (P < 0.01), Mental fatigue (P < 0.01)	Lower level of global side-effect burden(P<0.01)
Peppone et al. [27]	Breast	YC: 75 SC:92	Musculoskeletal symptoms in patients on endocrine therapy	FACIT-F, MFSI-SF	Significantly greater improvements in FACIT-F and MFSI-SF (P′S < 0.05)	Significantly greater reductions in the levels of pain, muscle aches, needing help finishing activities, time spent in bed, and feelings of heaviness in the body
Janelsins et al. [26]	Various (77% Breast)	YC: 168 SC:160	Memory difficulty	MDASI, General linear modelling	Decrease in memory difficulty (P<0 .05)	none
Lin et al. [25]	Various (77% Breast)	YC: 177 SC:181	CRF Sleep quality	MFSI-SF PSQI	Significantly greater improvements in CRF (P <0 .01), Sleep quality (P < 0.01), Subjective sleep quality (P=0.05), Daytime dysfunction (P< 0.01)	Significantly greater improvements in General (P < 0.01), physical (P < 0.01), emotional (P < 0.01), Mental fatigue (P < 0.01), Higher vigor (P <0.01)
Lin et al. [24]	Various (77% Breast)	YC: 176 SC:181	CRF impact on Walking Physical activity QoL	MDASI MFSI-SF Mediational analysis	Significant improvements in CRF’s interference with Walking (P=0.01), Physical activity (P<0.01), QoL (P<0.01)	

Cancer-Related Fatigue (CRF)

Sprod et al. conducted a multicentre, phase III, randomised controlled trial in older cancer survivors (≥60 years) [28]. Participants in the YOCAS group reported significantly lower levels of overall CRF, with notable improvements in physical, emotional, and mental fatigue compared with standard care. Lin et al. corroborated these findings in a larger cohort, demonstrating that YOCAS reduced CRF and improved survivors' engagement in physical activities and quality of life (QoL), suggesting a consequential effect of reduced fatigue on daily functioning [24].

Sleep Quality

Mustian et al. assessed sleep disturbances in 410 cancer survivors and found that it significantly improved global sleep quality compared to standard care [29]. Participants experienced improvements in sleep efficiency, waking after sleep onset, and reduced daytime dysfunction. Furthermore, the YOCAS participants reduced their use of sleep medications by 21% compared with 5% in the control group. Janelsins et al. found that improved sleep quality in the YOCAS group mediated reductions in memory difficulties, suggesting a beneficial indirect effect on cognitive function [26].

Cognitive Function and Memory

Janelsins et al. also directly assessed the impact of the YOCAS on cognitive function and reported a significant reduction in self-reported memory difficulties [26]. This study posited that improved sleep quality was a key factor in these cognitive improvements, highlighting the potential of YOCAS to ameliorate "chemobrain”, a common issue among cancer survivors.

Musculoskeletal Symptoms

Peppone et al. investigated YOCAS's impact of YOCAS on musculoskeletal pain in breast cancer survivors undergoing hormonal therapy [27]. YOCAS significantly reduced muscle aches, joint pain, and physical discomfort, with the most pronounced effects observed in the participants using aromatase inhibitors. This suggests that YOCAS may be an effective intervention for managing musculoskeletal side effects associated with hormonal therapy.

Summary of Results

Across the six clinical trials, the YOCAS programme consistently demonstrated significant reductions in cancer-related fatigue, addressing physical, emotional, and mental fatigue. Additionally, the participants experienced marked improvements in sleep quality, including enhanced sleep efficiency, reduced wakefulness, and lower dependence on sleep medications. Cognitive benefits were also observed, particularly reductions in self-reported memory difficulties, mediated by improved sleep quality. Furthermore, YOCAS was effective in reducing musculoskeletal pain, particularly among breast cancer survivors undergoing hormonal therapy.

Summary of Bias Assessment

**Figure 2:**
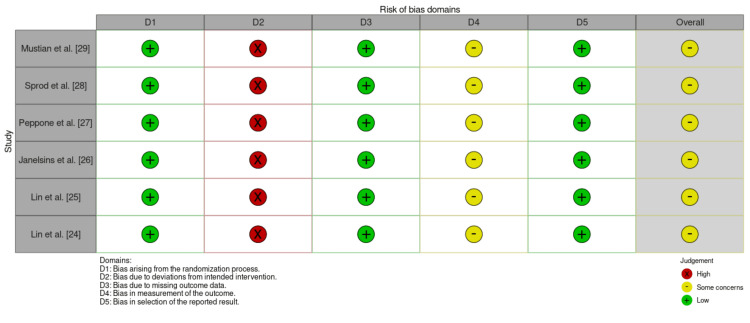
Risk of Bias Assessment for Included Studies Using the Cochrane RoB 2 Tool

The risk of bias for the included studies was evaluated using the Cochrane risk-of-bias tool. Most studies effectively minimised selection bias through computer-generated randomisation and stratified allocation concealment. However, due to the nature of the intervention, blinding of participants and personnel was not feasible, resulting in a high risk of performance bias. Some studies mitigated detection bias by blinding data analysts; however, those relying on self-reported outcomes, such as fatigue and sleep, remained vulnerable to bias. Attrition bias was low, with all studies reporting low dropout rates and utilising intention-to-treat analyses. Overall, the studies exhibited a low-to-moderate risk of bias, with robust randomisation and appropriate handling of missing data, supporting the reliability of the findings (Figure 2). 

Discussion

The findings from this systematic review underscore the positive impact of the YOCAS (Yoga for Cancer Survivors) programme on key cancer-related symptoms, including cancer-related fatigue (CRF), sleep quality, cognitive function, and musculoskeletal pain. YOCAS consistently demonstrated improvements in physical and psychological outcomes across the six reviewed trials, reinforcing its role as a valuable complementary therapy in survivorship care.

Compared to broader reviews that examine various yoga interventions, such as Niu et al. ’s recently published review, our review offers a more focused analysis of the standardised YOCAS program [30]. This standardised approach provides more robust evidence of YOCAS's specific benefits of YOCAS in addressing fatigue and improving sleep quality, two critical concerns for cancer survivors. While previous reviews have demonstrated the general effectiveness of yoga in improving the quality of life, it is distinguished by its consistent application and replicable results across studies. However, methodological challenges remain a common limitation in yoga studies, including the inability to blind the participants.

Cancer-related fatigue is a pervasive and debilitating symptom among cancer survivors. Both Lin et al. and Sprod et al. confirmed that YOCAS significantly reduced CRF, addressing the multidimensional aspects of fatigue, including the physical, emotional, and mental components [25,28]. Beyond merely reducing fatigue, the intervention led to improvements in the quality of life and physical activity, demonstrating its far-reaching effects on survivors' daily functioning.

Sleep disturbances are prevalent among cancer survivors and are often associated with psychological and physical challenges. Janelsins et al. and Mustian et al. demonstrated that YOCAS improved global sleep quality, reducing nocturnal wakefulness and dependence on sleep medications [26,29]. Furthermore, enhanced sleep quality indirectly mediates reductions in memory difficulties, highlighting the interconnectedness between sleep and cognitive function. This suggests that YOCAS, by improving sleep, may alleviate other cancer-related symptoms, such as cognitive impairment, commonly referred to as "chemobrain."

Cognitive impairment is a concern for cancer survivors, particularly for those who have undergone chemotherapy. Janelsins et al. reported significant improvements in self-reported memory difficulties following YOCAS participation [26]. While these improvements were not accompanied by objective cognitive testing, the perceived enhancement in memory function was significant for survivors, as cognitive symptoms are often subjectively experienced. The association between improved sleep and cognitive function highlights the broader neurocognitive benefits of targeting sleep through interventions such as YOCAS.

Musculoskeletal symptoms, including joint pain and muscle stiffness, are common side effects of hormonal therapy in breast cancer survivors. Peppone et al. demonstrated that YOCAS significantly reduced musculoskeletal pain and discomfort in this population, suggesting that the intervention could improve adherence to life-saving hormonal therapies by managing treatment-related side effects [27].

Clinical Implications

The consistent benefits of YOCAS across multiple studies suggest that it is a feasible, effective complementary therapy for cancer survivors. Clinicians can integrate YOCAS into post-cancer care by collaborating with Yoga Alliance-registered instructors who specialise in cancer-specific yoga to ensure patient safety and program efficacy. Establishing partnerships with local yoga studios, community centres, or oncology clinics can further enhance patient accessibility. One of the primary challenges in implementing the YOCAS program is the paucity of certified instructors with expertise in cancer care, particularly in certain regions. Establishing local training initiatives can address this deficit and expand access to qualified instructors. In addition, securing suitable community spaces and addressing the cost of YOCAS sessions, which are often not covered by insurance, can present obstacles. Offering group sessions or subsidised options could improve accessibility for a broader range of patients. To maximise the long-term benefits of YOCAS, healthcare providers should monitor patient adherence through regular assessments to ensure sustained engagement in the program.

Limitations

Although the results are promising, several limitations warrant consideration. The relatively short duration of YOCAS interventions (typically four weeks) constrains the understanding of the long-term sustainability of the benefits. Future studies should assess whether these improvements persist over extended follow-up periods and whether longer interventions yield greater or more durable effects. Furthermore, many studies relied on self-reported outcomes, which may have introduced a bias. Objective measures of sleep quality and cognitive function would provide more robust evidence of YOCAS's impact of YOCAS. Lastly, the heterogeneity of cancer types and stages across studies raises questions about the generalisability of the findings. Larger and more diverse data from newly published studies are necessary to confirm the efficacy of YOCAS across various cancer populations.

Future directions

Future research should focus on longitudinal studies to evaluate the durability of YOCAS benefits, as well as its impact on diverse cancer populations. Further investigation into the mechanisms underlying the effects of yoga on CRF, sleep, and cognitive function could help refine and tailor interventions for maximum benefits. Finally, exploring the cost-effectiveness of integrating YOCAS into survivorship care models could offer valuable insights for healthcare providers and policymakers who aim to adopt holistic treatment strategies.

## Conclusions

The YOCAS program offers significant benefits to cancer survivors, particularly in managing cancer-related fatigue, improving sleep quality, alleviating cognitive difficulties, and reducing musculoskeletal pain. Its low-risk, noninvasive nature renders it an advantageous option for enhancing survivorship care. As the field of integrative oncology continues to evolve, there is an increasing emphasis on non-pharmacological interventions, such as YOCAS, that address the holistic needs of cancer survivors. The positive outcomes observed in this review align with the broader movement towards integrating complementary therapies into conventional oncology care. YOCAS exemplifies how mind-body interventions can effectively address both the physical and psychological challenges faced by cancer survivors, offering a viable alternative or complement to pharmacological treatment. Future research should focus on expanding the accessibility of YOCAS, exploring its long-term benefits, and evaluating its cost-effectiveness in various healthcare settings. As the demand for personalised, patient-centred care increases, the role of yoga and similar interventions in survivorship care will likely continue to expand, contributing to the overall movement towards holistic, integrative cancer care.
